# Feasibility of in vivo swine models using guide wire-assisted intraductal radiofrequency ablation for benign biliary stricture

**DOI:** 10.1038/s41598-023-33867-9

**Published:** 2023-05-03

**Authors:** Jae Keun Park, Ju-Il Yang, Joo Kyung Park, Kwang Hyuck Lee, Jong Kyun Lee, Kyu Taek Lee

**Affiliations:** 1 Department of Internal Medicine, Kangnam Sacred Heart Hospital, Hallym University College of Medicine, Seoul, South Korea; 2 Division of Gastroenterology, Department of Medicine, Samsung Medical Center, Sungkyunkwan University School of Medicine, 81 Irwon-ro, Gangnam-gu, Seoul, 06351 South Korea

**Keywords:** Gastroenterology, Gastrointestinal diseases, Gastrointestinal models, Gastrointestinal system

## Abstract

Several in vivo swine models of benign biliary stenosis (BBS) have been recently reported for preclinical studies of novel endoscopic techniques and devices. The aim of this study was to evaluate the efficacy and feasibility of large animal models of BBS by using intraductal radiofrequency ablation (RFA) assisted by guide wire. Six in vivo swine models were made by using an intraductal RFA for cauterization at 10 W, 80 °C, 90 s in the common bile duct (CBD). Endoscopic retrograde cholangiopancreatography (ERCP) was performed with cholangiography and histologic evaluation was done for the common bile duct. Blood tests were examined before, after, and at the final follow-up. Guide wire assisted RFA electrode produced BBS in all (6/6, 100%) animal models without severe complications. Fluoroscopy findings at 2 weeks after intraductal RFA in every model revealed BBS in the common bile duct. In histologic evaluations, fibrosis and chronic inflammatory changes were noted. After the procedure, ALP, GGT, and CRP were elevated and decreased after an appropriate drain. A swine model of BBS is developed by inducing intraductal thermal injury using intraductal RFA assisted by guide wire. This novel technique for inducing BBS in swine is effective and feasible.

## Introduction

Endoscopic biliary drainage has been used as an initial treatment for benign biliary stricture (BBS). Recently, multiple plastic stents and covered self-expandable metal stents have been reported to show promising outcomes for the management of BBS^1,2^. However, adequate biliary drainage over the long-term using biliary stents is still challenging. Many biliary endoscopic devices including biliary stents have been recently developed to improve the patency of devices^3–5^. However, these attempts to increase the long-term drainage effect by changing the shape or material of plastic stents do not have satisfactory effects. Therefore, there are increasing requirements for new devices that can improve limitations of conducting endoscopic management in BBS.

Before applying to endoscopic devices in humans, appropriate animal models are important for preclinical study to evaluate the efficacy of endoscopic devices. In the past, previous studies mostly reported surgically created percutaneous approaches^6–8^. However, procedures of making animal models using surgically created percutaneous approaches were laborious. Recently, several studies have reported the development of in vivo and in vitro animal models for BBS using an endoscopic biliary approach^9–11^. The first endoscopic biliary approach by Rumella et al. used heat probe and multipolar probe to produce BBS^12^. However, these studies were conducted using methods including heat probe and multipolar probe other than intraductal radiofrequency ablation (RFA) for BBS. Even if an endoscopic approach was used, the experiment was conducted using a non-thermal injury method such as endoscopic detachable snare. Some studies have reported a short observation time without performing follow-up liver function tests commonly conducted in clinical practice.

Comparing other thermal injury methods for animal models of BBS, using intraductal RFA device is comfortable and easy to control energy dose (W), temperature (°C), and exposure time (sec). Using guide wire methods, intraductal RFA devices might more easily control the location and degree of bile duct injury. However, there were only a few investigations for intraductal application of RFA. In addition, an effective and safe energy dose for intraductal thermal injury with RFA is still not established. Thus, the aim of this study to assess the development of reproducible large animal models of BBS using endo biliary RFA and to investigate an effective and safe energy option for application in producing BBS.

## Results

### Fluoroscopic analysis via ERCP and blood analysis

**Figure 1:**
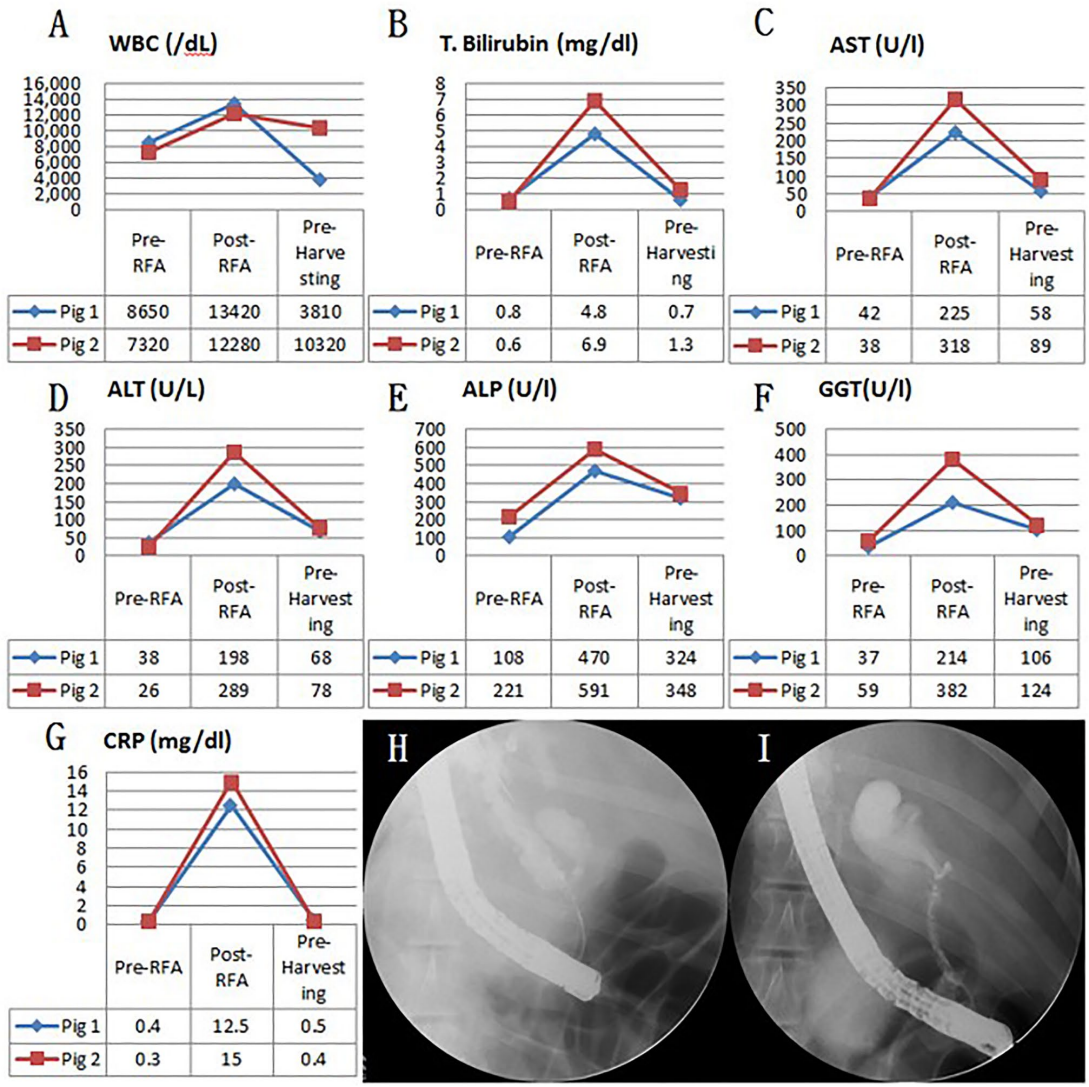
Blood test and cholangiography of biliary stricture pre-RFA, post-RFA, and at 1-month follow-up. (**A**) WBC changes; (**B**) T. bilirubin changes; (**C**) AST changes; (**D**) ALT changes; (**E**) ALP changes; (**F**) GGT changes; (**G**) CRP changes of 1 month follow-up pigs; (**H**) Cholangiography of biliary stricture at 1-month follow-up (pig 1); (**I**) Cholangiography of biliary stricture at 1-month follow-up (pig 2). *WBC* white blood cell count, *T. bilirubin* total bilirubin, *AST* aspartate transaminase, *ALT* alanine transaminase, *ALP* alkaline phosphatase, *GGT* gamma-glutamyl transferase, *CRP* C-reactive protein.

**Figure 2:**
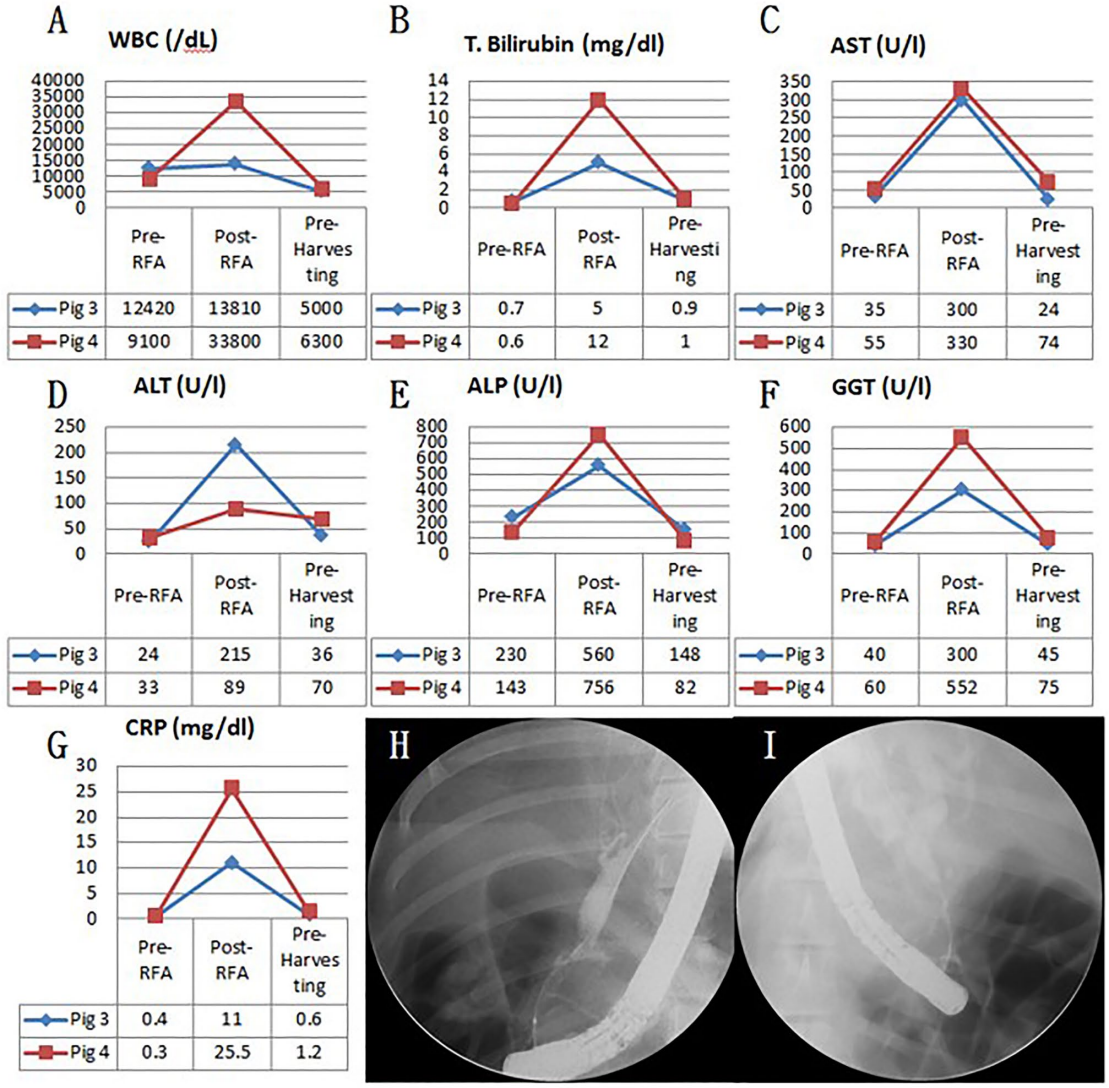
Blood test and cholangiography of biliary stricture pre-RFA, post-RFA, and at 3-month follow-up. (**A**) WBC changes; (**B**) T. bilirubin changes; (**C**) AST changes; (**D**) ALT changes; (**E**) ALP changes; (**F**) GGT changes; (**G**) CRP changes of 3-month follow-up pigs; (**H**) Cholangiography of biliary stricture of 3-month follow-up (pig 3); (**I**) Cholangiography of biliary stricture of 3-month follow-up (pig 4). *WBC* white blood cell count, *T. bilirubin* total bilirubin, *AST* aspartate transaminase, *ALT* alanine transaminase, *ALP* alkaline phosphatase, *GGT* gamma-glutamyl transferase, *CRP* C-reactive protein.

**Figure 3:**
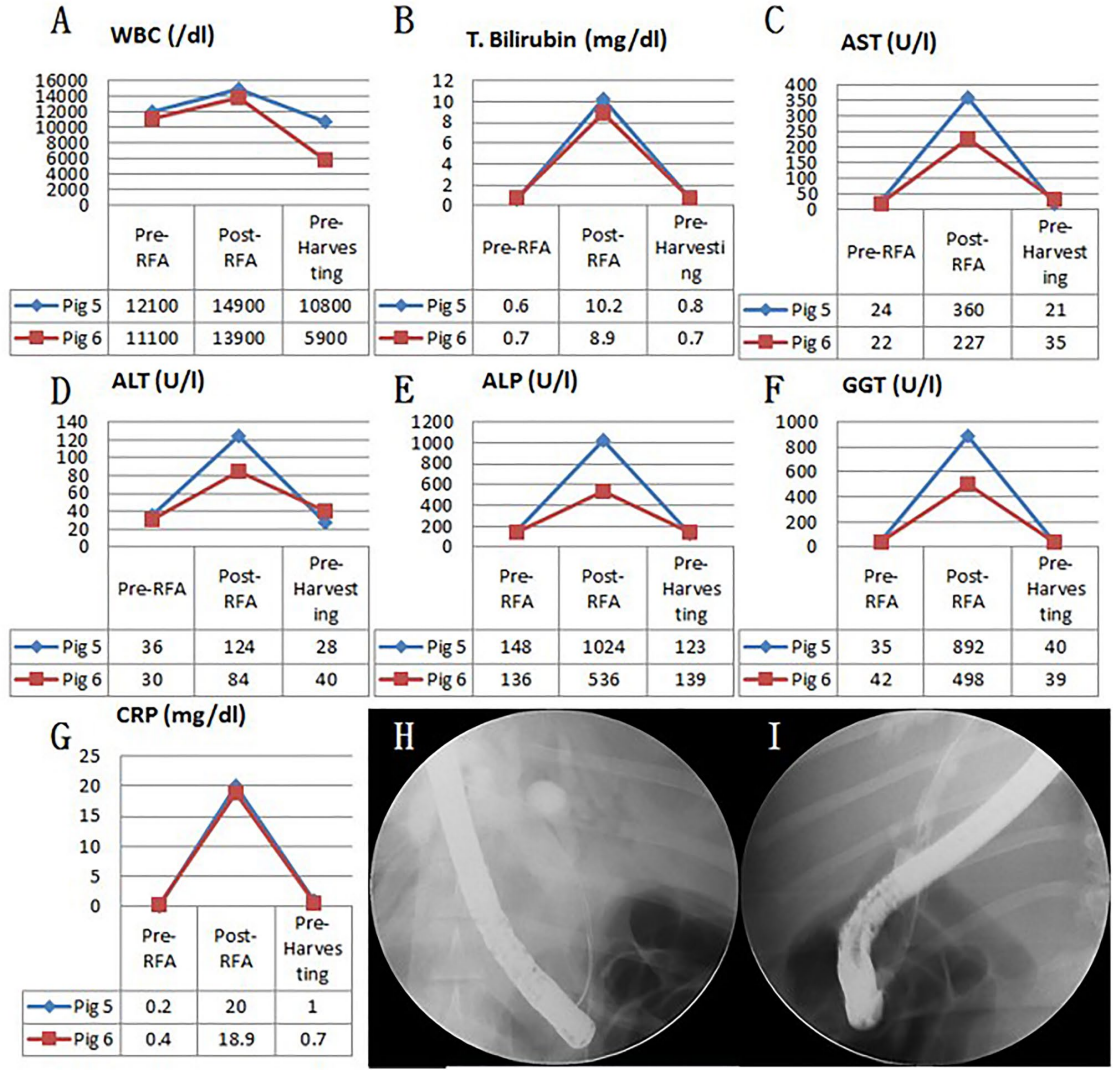
Blood test and cholangiography of biliary stricture pre-RFA, post-RFA, and at 5-month follow-up. (**A**) WBC changes; (**B**) T. bilirubin changes; (**C**) AST changes; (**D**) ALT changes; (**E**) ALP changes; (**F**) GGT changes; (**G**) CRP changes of 5-month follow-up pigs; (**H**) Cholangiography of biliary stricture of 5-month follow-up (pig 5); (**I**) Cholangiography of biliary stricture of 5-month follow-up (pig 6). *WBC* white blood cell count, *T. bilirubin* total bilirubin, *AST* aspartate transaminase, *ALT* alanine transaminase, *ALP* alkaline phosphatase, *GGT* gamma-glutamyl transferase, *CRP* C-reactive protein.

In all six swine animal models, we succeeded in generating BSS using intraductal RFA without any complications such as bleeding or perforation (success rate = 100% (6/6), severe complication rate = 0%). Blood levels of WBC, AST, ALT, ALP, GGT, and CRP of all experimental animals were measured before the intraductal RFA procedure, after the intraductal RFA procedure (2 weeks after RFA), and before euthanizing animals (Figs. 1, 2, 3A–G). Blood levels of WBC, AST, ALT, ALP, GGT, and CRP were elevated after the intraductal RFA procedure but decreased after biliary stenting. Figure 1H,I are biliary fluoroscopy findings at 2 weeks after RFA in experimental animals 1 and 2, demonstrating biliary stenosis. The same trend of blood test results as in Fig. 1 was observed in Fig. 2. The difference between Figs. 1 and 2 was that the follow-up was performed at 3 months after biliary stenting in Fig. 2. Biliary stenosis was confirmed by fluoroscopy findings at 2 weeks after RFA in experimental animals 3 and 4 (Fig. 2H,I). Experimental animals 5 and 6 were followed for 5 months after biliary stenting. They showed the same tendency of blood test results (Fig. 3A–G). Biliary stenosis was also confirmed by fluoroscopy findings at 2 weeks after RFA in experimental animals 5 and 6 (Fig. 3H,I).

### Macroscopic and microscopic histopathological evaluation

**Figure 4:**
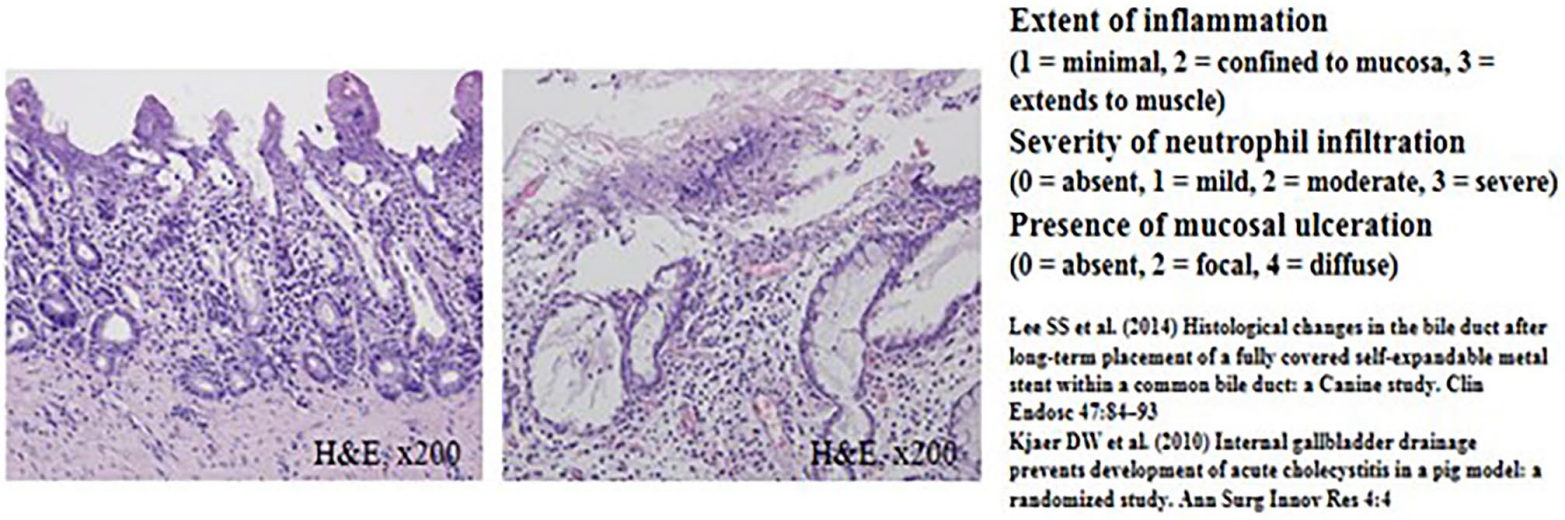
Comparison of microscopic images of bile duct inflammation using histologic scoring system between 1-month (left) and 3-month (right) follow-up animal models.

**Figure 5:**
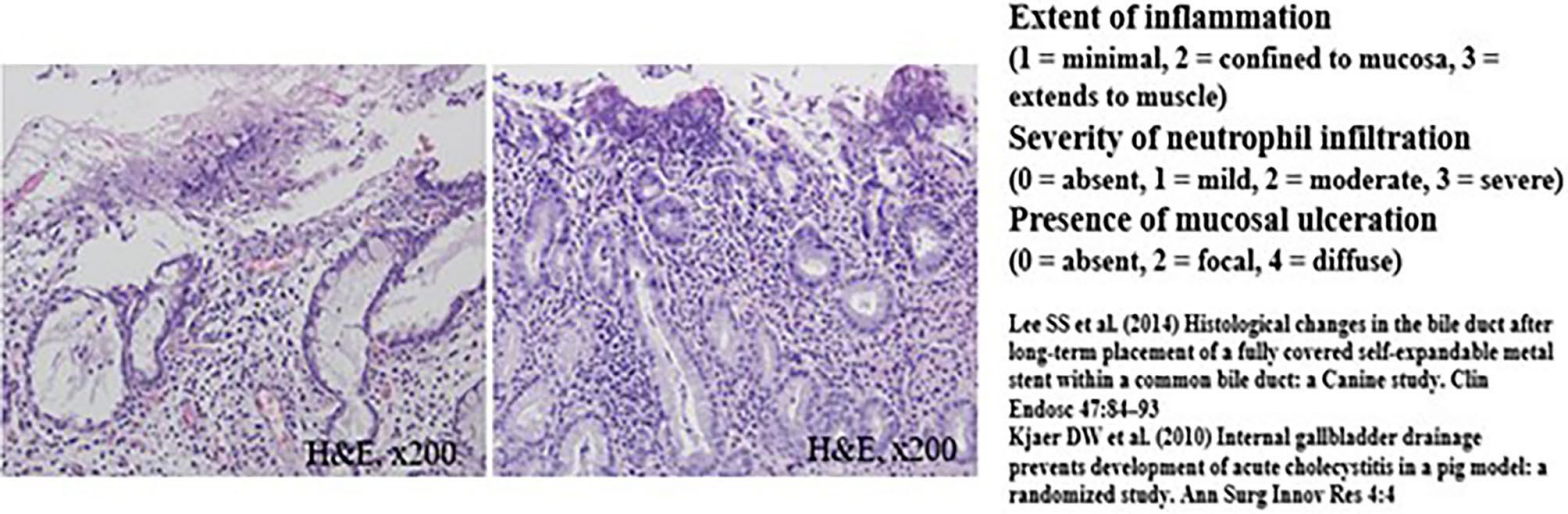
Comparison of microscopic images of bile duct inflammation using histologic scoring system between 3-month (left) and 5-month (right) follow-up animal models.

**Figure 6:**
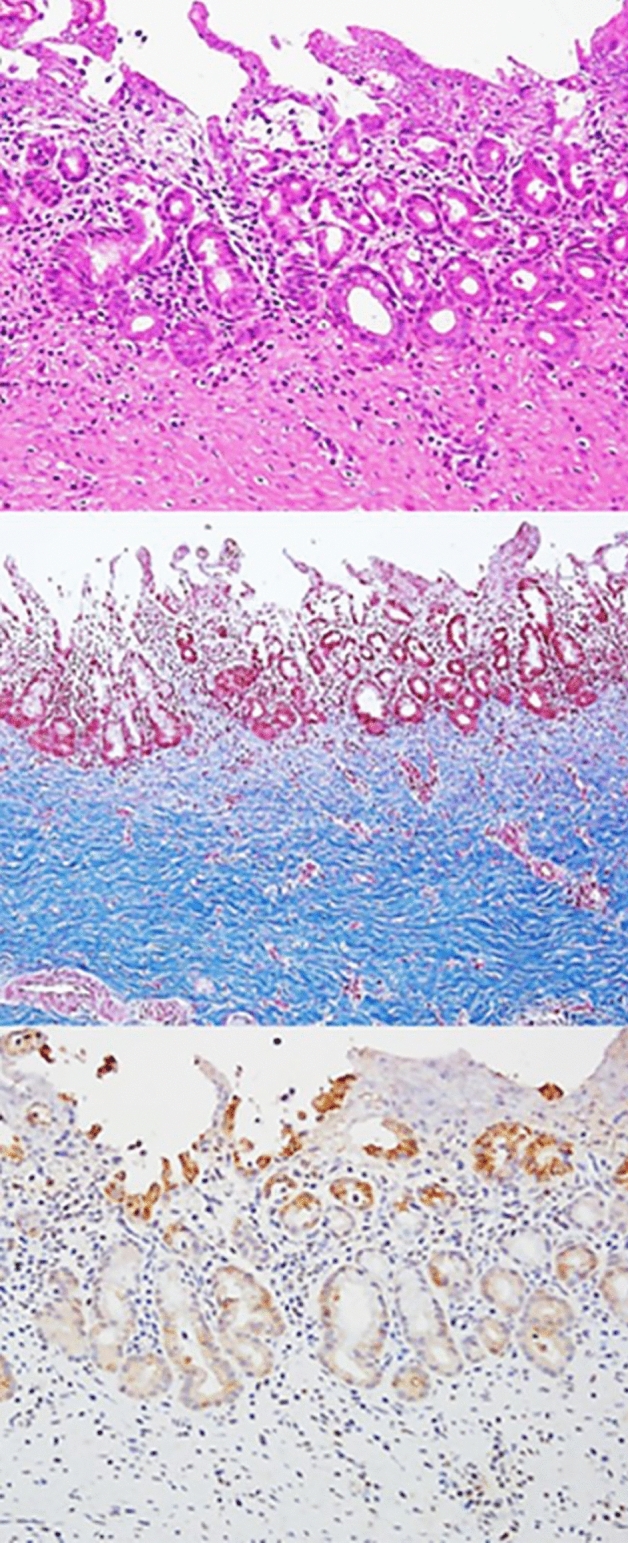
Microscopic images of plastic stented bile ducts from the swine animal model. H–E, M-T staining and CK 19 immunohistochemistry (from the top); *H–E* hematoxylin eosin, *M-T* Masson trichrome, *CK 19* cytokeratin 19.

The common bile duct (CBD) diameter was 2.5 ± 0.5 mm in macroscopic findings. The biliary stricture length measured after harvesting of experimental animals was 36 mm ± 0.5 mm in macroscopic findings. Using H&E-stained tissue sections, the degree of histological damage was compared by examining the depth of inflammation, the degree of deposition of neutrophils, the presence of mucosal ulceration, and the overall score (Figs. 4, 5 and Tables 1, 2). The total histological score indicating histological damage of plastic stents was observed to be higher for the 3-month point than that for the 1-month point total score, median (range) of 1-month pigs vs. 3-month pigs: 6 (6–8) vs. 6.5 (6–8), although their difference was not statistically significant (*p* = 0.057) (Fig. 4 and Table 1). However, in the animal group in which the plastic stents were mounted for 5 months, the total score was observed to be significantly higher than those mounted for less time (total score, median (range) of 3-month pigs vs. 5-month pigs: 6.5 (6–8) vs. 9 (9–9), *p* = 0.029) (Fig. 5 and Table 2). The degree of tissue damage was compared using immunofluorescent staining (Fig. 6). From the top to the bottom, photomicrographs of H&E, Masson Trichrome staining, and immunofluorescence staining using CK 19 are shown. H&E staining revealed that the deposition degree of neutrophils of the tissue in the contact area of the plastic stents (Fig. 6). When the same area was observed after Masson Trichrome staining, extensive fibrosis of the tissue in the contact area was observed. At the bottom of Fig. 6, the immunofluorescent staining was performed using CK 19 for histologic evaluation.

**Table 1:** Comparison of bile duct inflammation using histologic scoring system between 1- month and 3-month follow-up animal models.

	1 month	3 months	*P*
Extent of inflammation, median (range)	2 (2–2)	1.5 (1–2)	0.114
Severity of neutrophil infiltration, median (range)	2 (2–2)	2 (2–3)	0.114
Presence of mucosal ulceration, median (range)	2 (2–4)	3 (2–4)	0.686
Total score, median (range)	6 (6–8)	6.5 (6–8)	0.057

**Table 2:** Comparison of bile duct inflammation using histologic scoring system between 3- month and 5-month follow-up animal models.

	3 months	5 months	*P*
Extent of inflammation, median (range)	1.5 (1–2)	2 (2–2)	1.000
Severity of neutrophil infiltration, median (range)	2 (2–3)	3 (3–3)	0.029
Presence of mucosal ulceration, median (range)	3 (2–4)	4 (4–4)	0.114
Total score, median (range)	6.5 (6–8)	9 (9–9)	0.029

## Discussion

The aim of this study was to develop an in vivo swine model of BBS using guide wire assisted intraductal RFA. Animal experiments using in vivo bile ducts are important for preclinical tests during the development and improvement processes of pancreaticobiliary plastic stents technology. According to the literature, ERCP or laparoscopic surgery can be performed to reach the duodenal papilla and form a biliary stenosis model using detachable snare^11^. To form a biliary stenosis model, an intraductal heat probe through laparotomy or an intraductal radiofrequency heat therapy electrode can be used^10^. In this study, biliary stenosis models were successfully prepared using radiofrequency thermal therapy electrodes in the biliary tract through ERCP without post-procedure complications such as cholangitis or post ERCP pancreatitis. It is expected to be of great help in future preclinical research.

In this study, we performed H&E staining, Masson Trichrome staining, and immunofluorescence staining (CK 19) to achieve an objective histopathological evaluation. Furthermore, the veterinarian pathologist in the animal laboratory evaluated and calculated the histological score based on the histological scoring system shown in Supplementary Table 1 commonly used in previous studies^13–15^. Histological damage caused by plastic stents was found to be higher when the follow-up time was longer. Interestingly, the total histologic score did not show any significant differences between different time points less than 3 months after inserting biliary plastic stents. However, at 5 months after stent insertion, a statistically significant difference in histological score was observed compared to that at shorter follow-up. Considering our results and cost effectiveness of the animal study, 1-month point after RFA seems to be the best timing for testing the new devices in the BBS in vivo swine models. Therefore, in clinical practice, it is recommended to remove the plastic stent between 3 and 6 months, which is the average patency period in previous studies^16–18^ to avoid histological damage.

Our study has several limitations. First, this was a preclinical study using animal experiments. Results of the experiment cannot be generalized to human responses of patients with benign biliary stenosis disease. Second, the anatomy of bile ducts in swine is different from that in human. In swine, unlike human, the pancreatic duct and bile duct are separated. Finally, the efficacy and the feasibility were evaluated using a small number of experimental animals. Nevertheless, this study has its merit. It introduces the development of an experimental animal model of biliary stenosis with a reproducible, safe, and reliable method using radiofrequency heat therapy electrodes in the biliary tract.

In conclusion, the efficacy and safety of in vivo swine models of BBS using guide wire assisted intraductal RFA were confirmed through a preclinical laboratory animal study. In the future, a good method that can be used to evaluate the efficacy and safety of biliary plastic stents in the human body needs to be developed.

## Material and methods

### Preparation of experimental animals

A total of six female micro pigs (Micro pig M-type; Medi Kinetics Co., Ltd, Pyeongtaek, Gyeonggi-do, Korea) with an average weight of 50 kg at 10–12 weeks old were selected for animal experiments. Before starting the experiment, they were acclimated for 1 week. Only healthy animals were used for these experiments. All were bred in an animal breeding room set at a temperature of 23 ± 2 °C, a relative humidity of 50 ± 5%, a ventilation frequency of 10–12 times/hour, an illumination time of 08:00 to 20:00, and an illumination intensity of about 400 lx. During the quarantine period and experimental period, each pig was placed in one cage. Solid feed (Purina) was supplied twice a day, once before the start of day and once at 4:00 pm. Experiments were conducted after fasting for 24 h the day before the procedure. This study was carried out in accordance with relevant guidelines and regulation of the Animal Experimental Ethics Committee of the Samsung Life Science Research Institute, an accreditation body for AAALAC International (Association for Assessment and Accreditation of Laboratory Animal Care International) (IACUC Approval Number: 20160712001) and the Animal Research: Reporting of In Vivo Experiments guidelines (ARRIVE guidelines (https://arriveguidelines.org)) were followed for the in-vivo studies.

### Animal models for biliary stenosis using intraductal RFA

A total of six female micro pigs were randomly assigned to three groups (2 pigs per group) for 1 month, 3-month, and 5-month monitoring. The biliary stenosis model using electrode biliary cauterization was performed based on a previously published method^10^. Briefly, after fasting for 24 h, pigs were sedated by a veterinarian using ketamine^®^ 50 mg/ml 20 mg/kg, intramuscular injection of zolazepam (Zoletil^®^; 6 mg/kg), and xylazine (Rompun^®^; 2 mg/kg). Tracheal intubation was then performed. After tracheal intubation, anesthesia was maintained using 2% isoflurane. Electrocardiogram, heart rate, blood pressure, oxygen saturation, and end-tidal CO_2_ were monitored by the veterinarian. Enrofloxacin (2.5 mg/kg) was intramuscularly administered before the procedure and at 2 days after the procedure to prevent cholangitis due to the procedure. On the day of the procedure, ketoprofen (2 mg/kg) was intramuscularly administered for pain control. Endoscopic retrograde cholangiopancreatography (ERCP) was conducted using TJF240 (Olympus America, Inc, Melville, NY, USA), a therapeutic endoscope. First, we found and observed the duodenal papilla (Supplementary Fig. 1A). The ERCP was performed using the wire-guided cannulation method, which could selectively cannulate the bile duct using a guide wire under a fluoroscope (Supplementary Fig. 1B). After that, the Ampullae of Vater (AOV) was expanded with a Hurricane balloon catheter (Boston Scientific Corp., 10 mm in diameter) along the guide wire. The intra-biliary radiofrequency ablation (RFA) electrode was then inserted into the biliary tract along with the guide wire (Supplementary Fig. 1C,D). The RFA electrode (ELRA electrode, 7–10-W/33-mm type, STARmed Co. Ltd, Goyang, Gyeogi-do, Korea) mounted in the CBD was used for cauterization at 10 W and 80 °C for 90 s^19–21^. For maximizing the contact surface between the CBD and RFA electrode, the suction technique was used^21^.

### Confirmation of biliary stenosis in experimental animals and insertion of plastic stents

**Figure 7:**
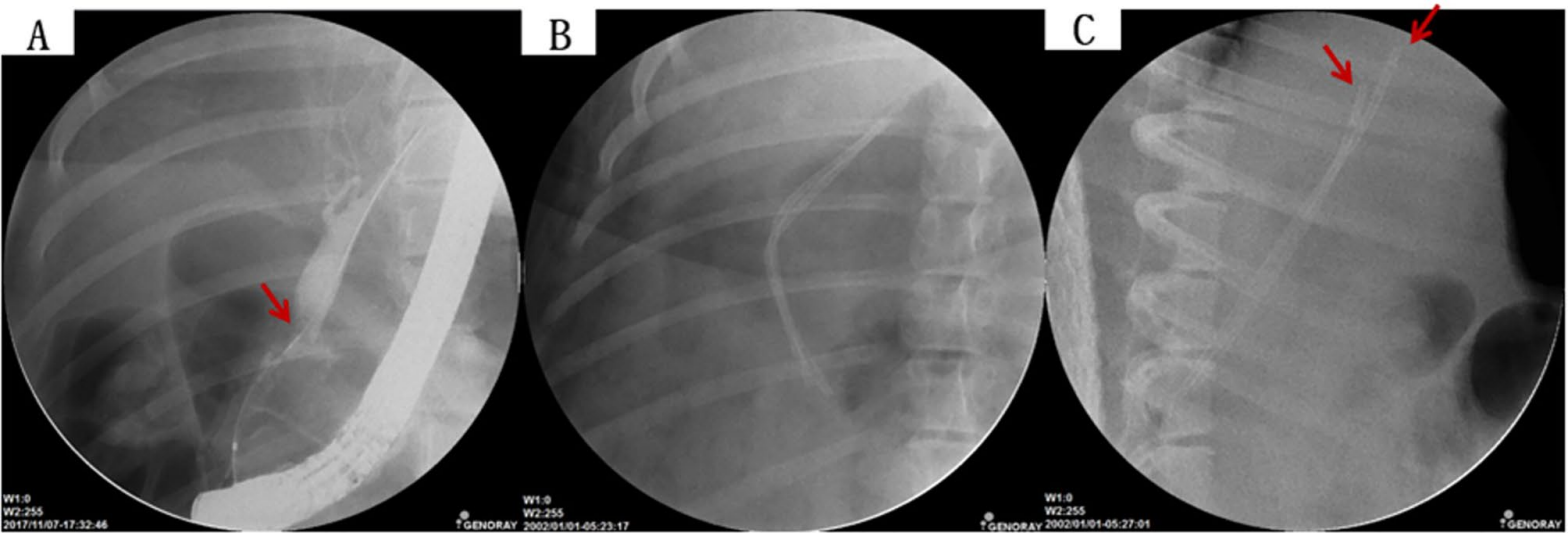
Fluoroscopy in swine models of biliary stenosis and plastic stent insertion. (**A**) Swine model of biliary stenosis (arrow) was confirmed by cholangiography images. Stenosis of the common bile duct with upstream dilatation; (**B**) Plastic stents were successfully inserted into swine bile duct; (**C**) Plastic stents were inserted into the dilated bile duct of swine. Proximal end of the plastic stent was placed in a different branch of the intra hepatic bile duct (arrows).

After 2 weeks of intraductal RFA, ERCP was performed to confirm biliary stenosis of treated experimental animals. Blood tests including White Blood Cell (WBC), Aspartate Transaminase (AST), Alanine Transaminase (ALT), Alkaline Phosphatase (ALP), Gamma-Glutamyl Transferase (GGT) and C-Reactive Protein (CRP) were performed before the procedure, after the procedure, and at the final follow-up. Using a 0.035-inch guidewire (Hydrophilic Tipped Guidewire, Boston Scientific Corp., Natick, MA, USA) under fluoroscopy, two commercially available 10-F biliary plastic stents (polyurethane tube of outer diameter 3.3 mm and inner diameter 2.0 mm; polyethylene tubing of outer diameter 3.3 mm and inner diameter 2.0 mm) were placed in the biliary tract (Fig. 7). The proximal end of the plastic stent was mounted to be located in the intrahepatic bile ducts of different branches (Fig. 7C).

### Harvesting of experimental animals

At 1 month, 3 months, and 5 months after plastic stents were inserted, 2 pigs at each time point were injected intramuscularly ketamine (Ketamine^®^; 50 mg/ml 20 mg/kg), zolazepam (Zoletil^®^; 6 mg/kg), and xylazine (Rompun^®^; 2 mg/kg) by a veterinarian on the same day of the procedure for sedation. After sedation, tracheal intubation was performed. Anesthesia was maintained using 2% isoflurane. Electrocardiogram, heart rate, blood pressure, oxygen saturation, and end-tidal CO_2_ were monitored by the veterinarian. Open laparotomy of all pigs was performed by one very skilled veterinarian. A median incision was made, and the duodenum was removed.

### Histopathological examination and evaluation

Pigs were euthanized at 1 month, 3 months, and 5 months after biliary plastic stents were inserted. Histopathological examination was then performed. After the liver, biliary tract, gallbladder, and duodenum were removed from experimental animals, a large amount of KCl (Potassium Chloride) was injected to induce euthanasia. From the extracted liver, biliary tract, gallbladder, duodenum, and AoV were dissected to the proximal end of the plastic stent located in the right and left intrahepatic bile duct. After the dissection, an incision was made in the longitudinal direction to confirm the proximal intrahepatic bile duct and distal biliary duct stenosis. The bile duct tissue was incised from the intrahepatic bile duct into which different plastic stents had been inserted and cut into sections, followed by H&E staining and Masson Trichrome staining. Histopathological examination was performed to reinforce the histological scoring method of previous studies. Scoring was performed in a blinded method using H&E-stained tissue sections. All sections were classified according to the extent of inflammation (1) (1, minimal; 2, confined to mucosa; and 3, extended to muscle with neutrophil infiltration). The severity of neutrophil infiltration was scored (2) as 0 for Absent, 1 for Mild, 2 for Moderate, and 3 for Severe. The presence of mucosal ulceration (3) was scored as 0 for Absent, 2 for Local, and 4 for Diffuse (Supplementary Table 1)^13–15^. Based on the histological scoring system shown in Supplementary Table 1, the histological score was calculated by a veterinarian pathologist in the animal laboratory of Samsung Life Sciences Research Institute.

### Statistical analysis

All experiments were performed or measured three or more times to compare the histopathology score. IBM SPSS version 24.0 (IBM Corp., Armonk, NY, USA) was used for all statistical analyses.

## Supplementary Information

Supplementary Information.

## Data Availability

The datasets of this study are available. All result generated or analyzed by the raw dataset are included in this published article and Supplementary Information files. Pus, the raw datasets generated during the study are available for research purposes from the corresponding author on reasonable request.

## Abbreviations

BBS: Benign biliary stenosis
RFA: Radiofrequency ablation
ERCP: Endoscopic retrograde cholangiopancreatography
WBC: White blood cell
AST: Aspartate transaminase
ALT: Alanine transaminase
ALP: Alkaline phosphatase
GGT: Gamma-glutamyl transferase
CRP: C-reactive protein
H–E: Hematoxylin eosin
M-T: Masson trichrome
CK 19: Cytokeratin 19
