# Supplementary material for: Feasibility of in vivo swine models using guide wire-assisted intraductal radiofrequency ablation for benign biliary stricture
Source: Sci Rep. 2023 May 3;13:7185. doi: 10.1038/s41598-023-33867-9 (PMC10156684; doi:10.1038/s41598-023-33867-9)

**Supplementary Material**

**Table 1.** The Histologic Score System Used for Determining the Severity of Inflammation of the Stented Bile Duct


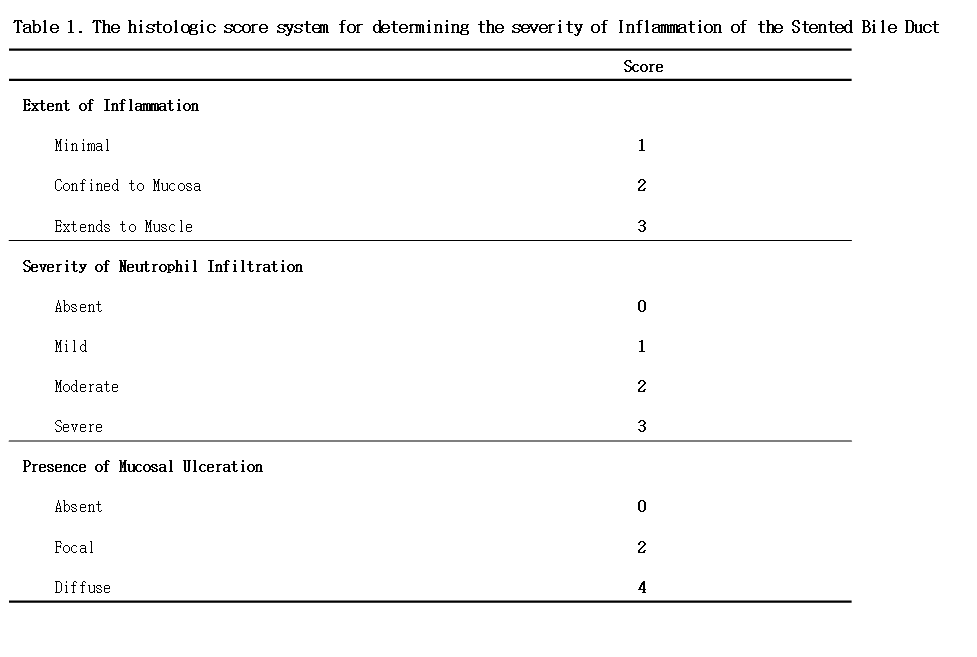


**Figure 1.** Preparation of Biliary Obstruction Animal Model Using Intraductal Radio Frequency Ablation (RFA).

A, Ampulla of Vater in Micro pig; B, Intraductal RFA electrode (ELRA electrode, STARmed Co. Ltd, Goyang, Gyeogi-do, Korea); C, Cholangiography of Micro pig before Intraductal RFA; D, Cholangiography of catheter with two radiologically marked electrodes


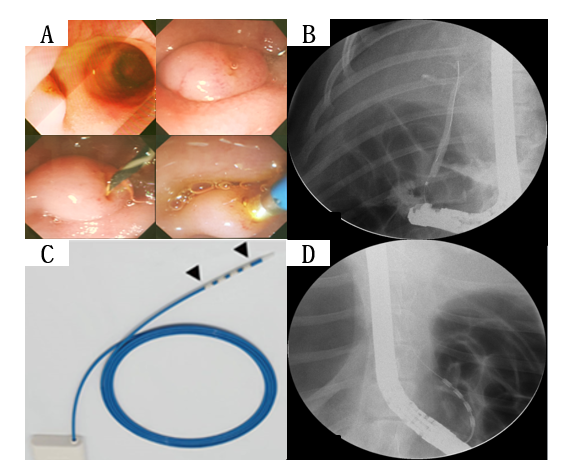

Supplement: Supplementary file 1 — Supplementary Information. [file 41598_2023_33867_MOESM1_ESM.docx]
